# On Multi-Hop Decode-and-Forward Cooperative Relaying for Industrial Wireless Sensor Networks

**DOI:** 10.3390/s17040695

**Published:** 2017-03-28

**Authors:** Yun Ai, Michael Cheffena

**Affiliations:** Faculty of Engineering, Norwegian University of Science and Technology (NTNU), N-2815 Gjøvik, Norway; michael.cheffena@ntnu.no

**Keywords:** wireless sensor network, cooperative relay, decode-and-forward, fading channel, harsh environment, impulsive noise, system performance, channel capacity, bit error rate

## Abstract

Wireless sensor networks (WSNs) will play a fundamental role in the realization of Internet of Things and Industry 4.0. Arising from the presence of spatially distributed sensor nodes in a sensor network, cooperative diversity can be achieved by using the sensor nodes between a given source-destination pair as intermediate relay stations. In this paper, we investigate the end-to-end average bit error rate (BER) and the channel capacity of a multi-hop relay network in the presence of impulsive noise modeled by the well-known Middleton’s class-A model. Specifically, we consider a multi-hop decode-and-forward (DF) relay network over Nakagami-*m* fading channel due to its generality, but also due to the absence of reported works in this area. Closed-form analytical expressions for the end-to-end average BER and the statistical properties of the end-to-end channel capacity are obtained. The impacts of the channel parameters on these performance quantities are evaluated and discussed.

## 1. Introduction

In recent years, technological concepts such as Industry 4.0, smart home, and smart grid are set to reshape the landscape of future industry and people’s lifestyle more than we ever thought possible. The common vision of such systems is generally connected to one single concept, the Internet of Things (IoT), where through the use of wireless sensor networks (WSNs), the entire physical infrastructure is closely coupled with the achievement of intelligent monitoring and management [1,2]. WSNs can provide great operating effectiveness through low installation and operating costs, installation flexibility, and scalability. They have be used in a broad range of scenarios such as smart home services [3], disaster detection and relief [4], and industrial automation [5,6].

A sensor network typically consists of a number of inexpensive low-power sensor nodes, which are distributed across a large area and can perform the tasks of data sensing, simple information processing, and communication over short distances [7]. Arising from the presence of spatially distributed sensor nodes in a sensor network, cooperative diversity can be achieved by using the sensor nodes between a given source-destination pair as intermediate relay stations. This kind of communication scheme provides significant robustness against the adverse effects of shadowing and fading in wireless communications, which leads to broader coverage, enhanced mobility, and improved system performance compared to the direct transmission [8,9,10]. Depending on the nature and complexity of the relaying technique, the relaying strategies can be generally classified into two categories, namely decode-and-forward (DF) and amplify-and-forward (AF) [11,12,13].

The performance analysis of DF and AF relaying systems under different channel conditions has been the topic of a wealth of papers. In the most recent work, the analytical expressions of the end-to-end average bit error rate (BER) for DF relaying systems over Rayleigh and Nakagami-*m* fading channels with additive white Gaussian noise (AWGN) have been derived in [14,15], respectively. The ergodic capacities of the DF relaying systems in Rayleigh, Rician, and Nakagami-*m* fading channels have been analyzed in [16,17,18], respectively. However, the analyses are made for the particular case of only two consecutive hops, which limits the use of the results. The ergodic capacity for multi-hop AF relaying systems over Rayleigh fading channels was investigated in [19]. In [20], the authors derived a tight lower bound for the error rate and the outage probability of cooperative diversity networks over independent non-identical Nakagami-m fading channels with AF relaying and maximum ratio combining (MRC) at the destination node. In [21], an upper bound for the ergodic capacity of multi-hop cooperative relaying channels over independent non-identically distributed (i.n.i.d.) Nakagami-m fading was derived assuming AF relays. From the aforementioned up-to-date reported works, it is fairly evident that the ergodic capacity for multi-hop DF relaying systems over Nakagami-*m* fading channels is not explored from the analytical point of view.

Meanwhile, the vast majority of the analyses on the performance of wireless communication systems in the open literature have been based on the assumption of interference amplitude following Gaussian distribution with flat power spectral density (i.e., AWGN) owing to its simplicity for analysis. However, the AWGN channel model does not cover the behavior of a large class of commonly occurring interference signals such as electromagnetic interference and man-made noises, which cannot be ignored in many scenarios ([22], pp. 84–90). For example, in harsh industrial environments where WSNs will play a vital role in the Industry 4.0 era, the effects of noise and interferences are significant due to the wide operating temperatures, strong vibrations and excessive electromagnetic noise caused by large motors and other equipment [23,24,25]. Therefore, the impulsive noise should be taken into consideration while analyzing the performance of WSNs for industrial applications. To this regard, a more practical model is the Middleton’s Class-A (MCA) model, which has shown to provide excellent fits to a variety of noise and interference measurements [26,27,28]. The advantage of this model lies in its generality to represent a number of interference signals with arbitrary impulsive effects. By varying model parameters, we can model a wide class of interferences ranging from pure AWGN to highly impulsive noise [29,30].

In light of this, we present the performance analysis of multi-hop DF relaying system over Nakagami-*m* fading channels in the presence of impulsive noise. The justification for the choice of Nakagami-*m* distribution as the small-scale fading in our analysis is threefold. Firstly, a large number of field measurements show that the small-scale fading in indoor environments follows Nakagami-*m* fading [31,32,33]. Secondly, Nakagami-*m* distribution describes via the *m* parameter a wide range of fading distributions. For instance, it converges to one-sided Gaussian distribution with m=1/2, to Rayleigh with m=1, and to purely Gaussian as *m* approaches infinity. Given appropriate bounds on the parameters, the lognormal and Weibull distributions can also be well approximated by the Nakagami-*m* distribution in some ranges ([34], pp. 284–288). Furthermore, it can also closely approximate the Nakagami-*n* (Rice) and Nakagami-*q* (Hoyt) distributions with appropriate parameter mappings ([35], p. 25). Thus, our results can be readily extended to other fading scenarios by simply varying the model parameters. Last but not least, to the best of our knowledge, it is still an open research question on the capacity performance of multi-hop DF relaying systems over Nakagami-*m* fading channels. In this paper, we intend to fill this gap.

The remainder of this paper is organized as follows. In Section 2, we introduce the cooperative transmission system under investigation as well as the fading channel and noise model. In Section 3, closed-form expressions for the end-to-end average BER of the relaying system under the considered channel condition are derived. The analytical expression for the end-to-end average capacity and the statistics of the end-to-end instantaneous capacity are derived in Section 4. Analytical and simulation results are presented and discussed in Section 5. Section 6 concludes the paper and discusses about future work.

## 2. System and Channel Model

### 2.1. Notation

A number of notations are used throughout the paper, here we highlight the following notations and their corresponding meanings: $f_{x}\left(\cdot \right)$ denotes the probability density function (PDF) of the random variable (RV) *x*; $F_{x}\left(\cdot \right)$ represents the cumulative distribution function (CDF) of the RV *x*. The symbols $P_{Tx,\ell }$ and $P_{Rx,\ell }$ are the transmitted power and the received signal power by node $R_{\ell }$, respectively. The RV $z_{\ell }$ represents the noise of the *ℓ*-th link modelled by MCA model. The symobl $P_{\ell }$ represents the average BER of the *ℓ*-th hop transmission; and $P_{\ell }^{E}$ denotes the average BER after the *ℓ*-th transmission compared with the bits transmitted by source node $R_{1}$.

### 2.2. System Model

**Figure 1:**
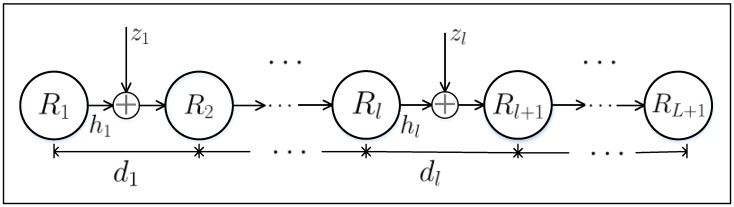
Illustration of a multi-hop relay system with *L* hops.

In our work, we consider a multi-hop wireless relay system with L hops using DF relaying strategy. Figure 1 illustrates the investigated scenario, where the nodes $R_{1}$ and $R_{L+1}$ correspond to the source node and destination node, respectively; and the link between the nodes $R_{\ell }$ and $R_{\ell +1}$, ℓ=1,…,L, is denoted as *ℓ*-th hop with separation distance $d_{\ell }$. Full-duplex mode of communication is assumed, in which the nodes can transmit and receive at the same time employing frequency division duplexing (FDD). In FDD mode, each node $R_{\ell }$ use frequency bands $B_{\ell }^{u}$ and $B_{\ell }^{d}$ to transmit and receive data, respectively. Altogether, there are *L* such non-overlapping frequency pairs; and only a subsequent node can listen to the signal transmitted by a previous node, thus avoiding the interference in any hop owing to the transmissions occurring in the surrounding hops. The channels between adjacent nodes $R_{\ell }$ and $R_{\ell +1}$, ℓ=1,…,L, are mutually independent and undergo Nakagami-*m* fading.

Data transmission is done using the M-ary phase shift keying (M-PSK) symbols with equal a priori probabilities. The node $R_{\ell }$, ℓ=1,…,L, transmits the unit-energy M-PSK symbol $s_{\ell }$ (i.e., $E_{b}\;=\;E[|s_{\ell }{|}^{2}]=1$) with power $P_{Tx,\ell }$. The corresponding received signal $r_{\ell +1}$ at node $R_{\ell +1}$ and the total transmit power $P_{Tx}$ of the relay system can be written as

(1) $$\begin{array}{cc} r_{\ell +1} & =\sqrt{P_{Rx,\ell +1}}\cdot h_{\ell }\cdot s_{\ell }+z_{\ell },\;\;\;\;\;\ell =1,\ldots ,L \end{array}$$

(2) $$\begin{array}{cc} P_{Tx} & =\sum_{\ell =1}^{L}P_{Tx,\ell }, \end{array}$$

where $P_{Rx,\ell +1}$ is the received signal power by node $R_{\ell +1}$; $h_{\ell }$ and $z_{\ell }$ are the channel fading amplitude and the additive noise of the *ℓ*-th hop, respectively. The received signal power $P_{Rx,\ell +1}$ by node $R_{\ell +1}$ depends on the transmitted signal power $P_{Tx,\ell }$ by the previous node $R_{\ell }$ and the distance $d_{\ell }$ between the two nodes. A widely used model for the signal attenuation is the one-slope path-loss model [33]. According to the one-slope model, the relationship between $P_{Rx,\ell +1}$ and $P_{Tx,\ell }$ can be expressed as

(3) $$10\log_{10}\left(P_{Rx,\ell +1}\right)=10\log_{10}\left(P_{Tx,\ell }\right)-10\cdot \xi \cdot \log_{10}\left(d_{\ell }\right),\;\;\;\;\;\ell =1,\ldots ,L$$

where ξ is the path-loss exponent, which reflects the rate at which the received power decreases with distance. The value of the parameter ξ in a specific environment could be simply obtained by field measurement [36,37,38] or estimated using numerical algorithms [39,40]. The overall performance of the DF relaying network is limited by the worst link, it is thus straightforward to show that in order to obtain the best performance, the total power should be allocated among different hops in a way such that the received signal power is equal for each receiving node, i.e., $P_{Rx,\ell }=P_{Rx,\ell +1}=P_{Rx}$, ℓ=2,…,L. From (3), this constraint of the received powers translates into the following relationship on the transmitted power of each node

(4) $$\frac{P_{Tx,1}}{{\left(d_{1}\right)}^{\xi }}=\frac{P_{Tx,2}}{{\left(d_{2}\right)}^{\xi }}=\ldots =\frac{P_{Tx,L}}{{\left(d_{L}\right)}^{\xi }}.$$

### 2.3. Channel Model

The channel fading amplitudes $h_{\ell }$ (ℓ=1,…,L) in (1) are modelled as independent and identically distributed (i.i.d.) Nakagami-*m* RVs. The PDF $f_{h_{\ell }}\left(h\right)$ of the RVs $h_{\ell }$ (ℓ=1,…,L) is expressed as

(5) $$f_{h_{\ell }}\left(h\right)=\frac{2m^{m}h^{2m-1}}{\Omega^{m}\Gamma \left(m\right)}\cdot \exp\left(-\frac{mh^{2}}{\Omega }\right),\;\;\;\;\;h\geq 0$$

where Γ(·) represents the complete Gamma function defined as $\Gamma \left(\tau \right)=\int_{0}^{\infty }t^{\tau -1}\cdot \exp\left(-t\right)\;dt$, $\Omega \;=\;E[|h_{\ell }{|}^{2}]$ is the expectation of $|h_{\ell }{|}^{2}$; and *m* is the Nakagami-*m* fading parameter, which determines the severity of fading channels and ranges from 0.5 to ∞.

The additive noise $z_{\ell }$ (ℓ=1,…,L) in (1) are i.i.d. RVs modeled by the MCA model. The PDF $f_{z_{\ell }}\left(z\right)$ of the RVs $z_{\ell }$ is given by [41]

(6) $$\begin{array}{cc} f_{z_{\ell }}\left(z\right)= & \sum_{n=0}^{\infty }\frac{\alpha_{n}}{\sqrt{2\pi }\;\sigma_{n}}\cdot \exp\left(\frac{-z^{2}}{2\sigma_{n}^{2}}\right) \end{array}$$

(7) $$\begin{array}{cc} = & \sum_{n=0}^{\infty }\alpha_{n}\cdot N\left(z;0,\sigma_{n}\right), \end{array}$$

where $N(z;0,\sigma_{n})$ denotes a zero-mean Gaussian PDF with variance $\sigma_{n}^{2}$; and the parameter $\alpha_{n}$ is the Poisson-distributed probability expressed by

(8) $$\alpha_{n}=\frac{\exp\left(-A\right)\cdot A^{n}}{n!}.$$

**Figure 2:**
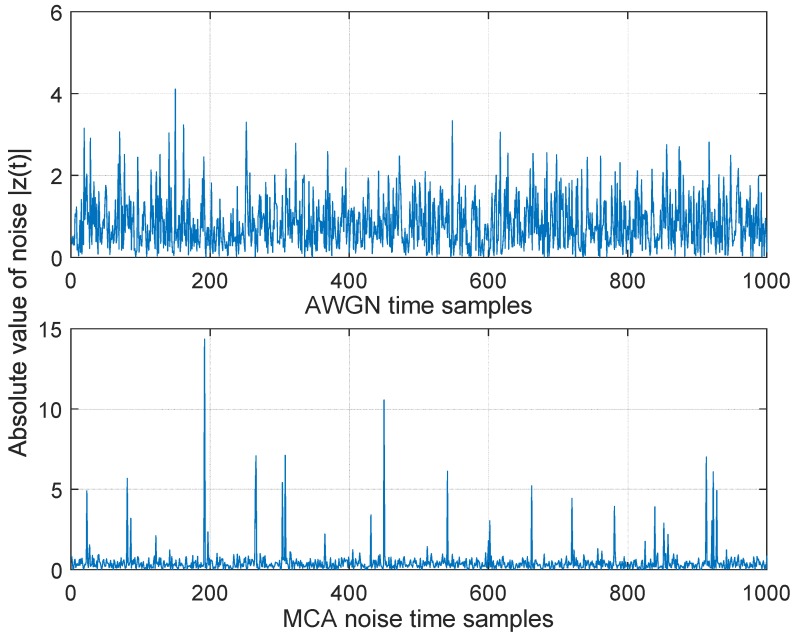
Absolute values of AWGN samples with $N_{0}=1$ (**top** figure) and Middleton’s Class-A noise samples with $N_{0}=1$, A=0.01, ρ=0.1 (**bottom** figure).

**Figure 3:**
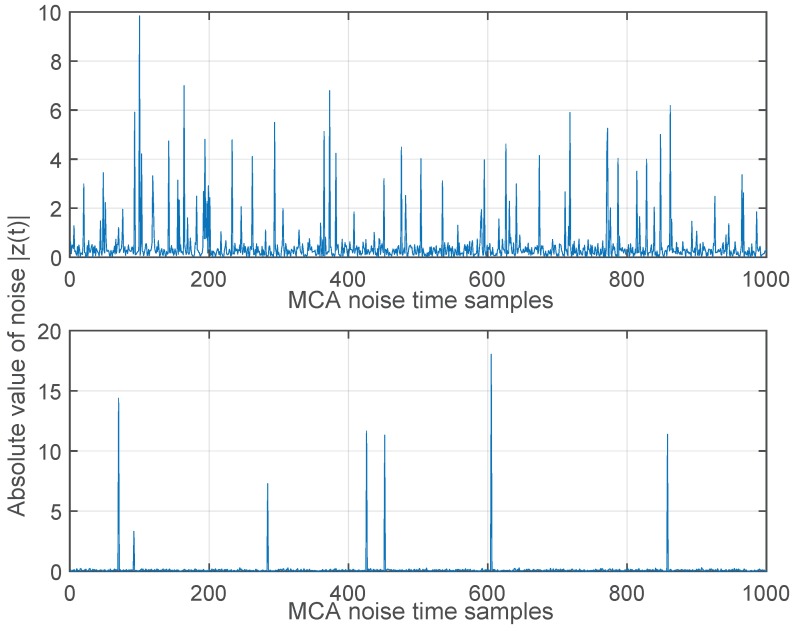
Absolute values of Middleton’s Class-A noise samples with $N_{0}=1$, A=0.1, ρ=0.1 (**top** figure) and with $N_{0}=1$, A=0.01, ρ=0.01 (**bottom** figure).

The variance $\sigma_{n}^{2}$ in (6) and (7) is defined with the auxiliary RV $\beta_{n}$ as follows:

(9) $$\begin{array}{cc} \sigma_{n}^{2} & =\left(\sigma_{g}^{2}+\sigma_{i}^{2}\right)\cdot \beta_{n} \end{array}$$

(10) $$\begin{array}{cc} \beta_{n} & =\frac{\left(n+A\rho \right)}{A\cdot \left(1+\rho \right)}, \end{array}$$

where ρ represents the ratio of the Gaussian noise power $\sigma_{g}^{2}$ to the impulsive noise power $\sigma_{i}^{2}$. The parameter *A* is called the impulsive index and defined as the product of average rate of impulsive noise and mean duration of the impulsive interference. The impulsive index determines impulsiveness the noise: a smaller value of the impulsive index implies a higher level of impulsive interference. It is known that as *A* approaches a value around 10 or larger, the MCA distribution is very close to a Gaussian PDF; while for *A* and ρ lower than 1, the PDF gets very heavy tails and the interference can be seen as very impulsive [26]. The extraction of MCA model parameters from real measurements can be done with the algorithms developed in (e.g., [42,43]). It should be noted that the MCA model includes both the impact of impulsive interference as well as thermal noise in the communication systems [41]. Figure 2 shows the difference between pure AWGN and impulsive noise generated with the MCA model and Figure 3 illustrates the effects of the parameters *A* and ρ used in the MCA model.

As can be seen from (7), the MCA model can be interpreted as a Gaussian-mixture model [44]. Therefore, it is straightforward to show that the mean of the noise $u_{z_{\ell }}=E\left[z_{\ell }\right]=0$; and the average power $N_{0}$ of the noise, i.e., the variance of $z_{\ell }$, is calculated as

(11) $$\begin{array}{cc} N_{0} & =E\left[z_{\ell }^{2}\right]=\sum_{n=0}^{\infty }\frac{\exp\left(-A\right)\cdot A^{n}}{n!\sqrt{2\pi }\sigma_{n}}\int_{-\infty }^{\infty }z^{2}\cdot \exp\left(-\frac{z^{2}}{2\sigma_{n}^{2}}\right)\;dz \end{array}$$

(12) $$\begin{array}{c} =\frac{\exp\left(-A\right)\cdot \sigma_{g}^{2}}{\rho }\cdot \sum_{n=0}^{\infty }\frac{A^{n}}{n!}\cdot \left(\frac{n}{A}+\rho \right)=\sigma_{g}^{2}+\sigma_{i}^{2}. \end{array}$$

Alternatively, the generation of MCA noise can be interpreted as a stationary random process: for each channel realization, the probability of experiencing an interference noise with distribution $N(z;0,\sigma_{n})$ (n=0,…,∞) is $\alpha_{n}$ given in (8). Despite the PDF in (7) has an infinite number of terms, we can safely restrict our analysis to the first *N* terms without significant loss by noticing that as *n* increases, the probability $\alpha_{n}$ approaches zero and is thus negligible. The number *N* can be obtained from

(13) $$N=\arg\min_{N}\;\left(1-\sum_{n=0}^{N}\alpha_{n}\right)\leq \epsilon ,$$

where ϵ is an arbitrary positive number.

## 3. Bit Error Rate Performance Analysis

### 3.1. The Instantaneous SNR

To derive the BER and channel capacity of the relaying system, we first derive the statistics of the signal-to-noise ratio (SNR). Let $\gamma_{\ell }$ be the instantaneous SNR pertaining to the *ℓ*-hop transmission, which is given by

(14) $$\gamma_{\ell }=\frac{P_{Rx,\ell +1}\cdot h_{\ell }^{2}\cdot E_{b}}{N_{0}}.$$

As the fading amplitude $h_{\ell }$ is Nakagami-*m* distributed with PDF given in (5), we can obtain the PDF $f_{\gamma_{\ell }}\left(\gamma \right)$ of the instantaneous SNR $\gamma_{\ell }$ by applying the concept of transformation of random variables ([45], pp. 182–193) and obtain the following expression:

(15) $$f_{\gamma_{\ell }}\left(\gamma \right)=\frac{{\left(mN_{0}\right)}^{m}\cdot \gamma^{m-1}}{{\left(\Omega E_{b}\cdot P_{Rx,\ell +1}\right)}^{m}\cdot \Gamma \left(m\right)}\cdot \exp\left(-\frac{mN_{0}\gamma }{\Omega E_{b}\cdot P_{Rx,\ell +1}}\right)\;\;\;\;\;\gamma \geq 0.$$

From (15), we could also obtain the average SNR ${\bar{\gamma }}_{\ell }$ of the *ℓ*-th hop as ${\bar{\gamma }}_{\ell }=\left(\Omega E_{b}\cdot P_{Rx,\ell +1}\right)/N_{0}$. The CDF $F_{\gamma_{\ell }}\left(\gamma \right)$ of the instantaneous SNR $\gamma_{\ell }$ can be obtained from its relationship with the PDF $f_{\gamma_{\ell }}\left(\gamma \right)$ in (15) as follows:

(16) $$\begin{array}{cc} F_{\gamma_{\ell }}\left(\gamma \right) & =\frac{{\left(mN_{0}\right)}^{m}}{{\left(\Omega E_{b}\cdot P_{Rx,\ell +1}\right)}^{m}\cdot \Gamma \left(m\right)}\int_{0}^{\gamma }\gamma^{m-1}\cdot \exp\left(-\frac{mN_{0}}{\Omega E_{b}\cdot P_{Rx,\ell +1}}\gamma \right)\;d\gamma \\ & =\tilde{\Gamma }\left(m,\frac{mN_{0}}{\Omega E_{b}\cdot P_{Rx,\ell +1}}\gamma \right), \end{array}$$

where $\tilde{\Gamma }\left(\cdot ,\cdot \right)$ is the normalized lower incomplete Gamma function defined by

(17) $$\tilde{\Gamma }\left(\tau ,y\right)=\frac{1}{\Gamma (\tau )}\int_{0}^{y}t^{\tau -1}\cdot \exp\left(-t\right)\;dt.$$

### 3.2. The End-to-End Average BER

**Proposition** **1.** *The average BER $P_{\ell }$ of the M-ary phase shift keying (M-PSK) for the ℓ-th hop over the Nakagami-m fading channel in the presence of impulsive noise is given by*

(18) Pℓ=∑n=0∞exp(−A)·Ann!·(m·N0)2m(ΩEb·PRx,ℓ+1)2m·1ζM∑ι=1max(M4,1)[1−2A(1+ρ)mN0·ηι2π(n+Aρ)ΩEb·PRx,l+1·ΩEbPRx,ℓ+1m·N0(m+12)·F121,m+12;32;A(1+ρ)ΩEbPRx,ℓ+1·ηι22mN0(n+Aρ)+ΩEbPRx,ℓ+1A(1+ρ)·Γ(m+12)Γ(m)·1mN0ΩEbPRx,ℓ+1+A(1+ρ)ηι22(n+Aρ)m+12],

*where $\zeta_{M}=\max\left(\log_{2}M,2\right)$, $\eta_{\iota }=\sin\left(\frac{(2\iota -1)\cdot \pi }{M}\right)$ and _2_$F_{1}\left(a,b;c;x\right)$ represents the Gauss hypergeometric function ([46], p. 1005) defined by*

(19) F12(a,b;c;x)=∑p=0∞(a)p(b)p(c)pxpp!,

*with ${\left(g\right)}_{p}=g\left(g+1\right)\ldots \left(g+p-1\right)$ denoting the ascending factorial.*

**Proof.**  See Appendix A.1. ☐

**Remark.**  *The average BER $P_{\ell }$ of equiprobable binary phase shift keying (BPSK) modulated symbols for the ℓ-th hop over the Nakagami-m fading channel in the presence of impulsive noise can be readily obtained from (18) as follows:*

(20) Pℓ=∑n=0∞exp(−A)·An2n!·(m·N0)2m(ΩEbPRx,ℓ+1)2m·[1−2A(1+ρ)mN0π(n+Aρ)ΩEbPRx,ℓ+1·Γ(m+12)Γ(m)·ΩEbPRx,ℓ+1m·N0(m+12)F121,m+12;32;A(1+ρ)ΩEbPRx,ℓ+12mN0(n+Aρ)+ΩEbPRx,ℓ+1A(1+ρ)·1mN0ΩEbPRx,ℓ+1+A(1+ρ)2(n+Aρ)m+12].

**Proposition** **2.** *If the average BER per hop is the same for all hops (namely, $P_{\ell }=P_{0}$, ℓ=1,⋯,L), then the end-to-end average BER $P_{L}^{E}$ of L-hop the wireless DF relaying system can be expressed as:*

(21) $$\begin{array}{c} P_{L}^{E}=\frac{1}{2}\left[1-{\left(1-2P_{0}\right)}^{L}\right]. \end{array}$$

**Proof.**  See Appendix A.2. ☐

**Figure 4:**
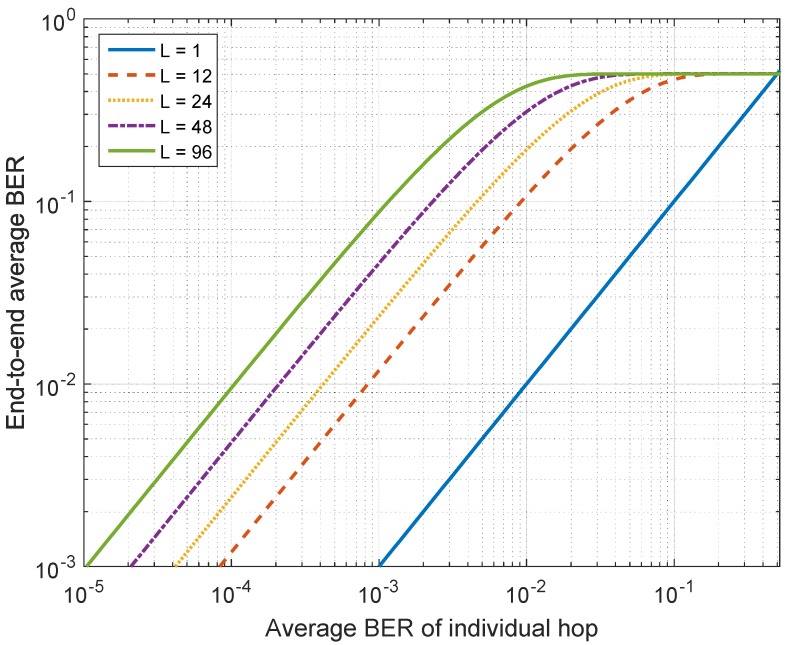
End-to-end average BER of the *L*-hop relay network versus individual-hop average BER.

Figure 4 shows the relationship between the end-to-end average BER of the *L*-hop relay system and the single-hop average BER in log-log representation assuming the identical statistical behavior for all single hops. From Figure 4, we can observe a nearly linear relationship between the two BERs when the single-hop average BER is below some threshold. This linearity can be theoretically proved by noticing that the linear term will dominate for low values of $P_{0}$ after extending the power series in (21) ([46], p. 25).

## 4. Channel Capacity Performance Analysis

By comprehending the MCA noise channel as a Gaussian-mixture channel in (7), the instantaneous channel capacity per unit bandwidth $C_{\ell }\left(\gamma \right)$ of the *ℓ*-th link can be obtained according to the theorem of total probability ([45], p. 23), i.e.,

(22) $$C_{\ell }\left(\gamma \right)=\sum_{n=0}^{\infty }\alpha_{n}\cdot \log_{2}\left(1+\frac{\gamma }{\beta_{n}}\right),$$

where $\alpha_{n}$ and $\beta_{n}$ are given in (8) and (10), respectively.

According to the well-known max-flow min-cut theorem, the maximum achievable channel capacity of a multi-hop relay system from the source node to the destination node is bounded by the minimum of the capacities of each individual hop ([47], pp. 587–595). Therefore, the instantaneous channel capacity $C(\gamma_{e})$ of the end-to-end full-duplex relay network can be written as

(23) $$C\left(\gamma_{e}\right)=\min_{\ell =1,\ldots ,L}\left\{C_{\ell }\left(\gamma \right)\right\}=\sum_{n=0}^{\infty }\frac{\alpha_{n}}{\ln(2)}\cdot \ln\left(1+\frac{\gamma_{e}}{\beta_{n}}\right),$$

where $\gamma_{e}=\min\left\{\gamma_{1},\ldots ,\gamma_{L}\right\}$ is the effective SNR equivalent to one-hop transmission channel with the same capacity as the *L*-hop DF relay channel.

**Proposition** **3.** *The PDF $f_{\gamma_{e}}\left(\gamma \right)$ of the equivalent SNR $\gamma_{e}$ of the L-hop relay system is expressed as*

(24) $$f_{\gamma_{e}}\left(\gamma \right)=\frac{L}{\Gamma (m)}\cdot {\left[\hat{\Gamma }\left(m,\frac{mN_{0}}{\Omega E_{b}P_{Rx,\ell +1}}\gamma \right)\right]}^{L-1}\cdot {\left(\frac{mN_{0}}{\Omega E_{b}P_{Rx,\ell +1}}\right)}^{m}\cdot \gamma^{m-1}\cdot \exp\left(-\frac{mN_{0}}{\Omega E_{b}P_{Rx,\ell +1}}\gamma \right).$$

**Proof.**  Under the power allocation scheme in (4), the instantaneous SNR $\gamma_{\ell }$ (ℓ=1,…,L) of each hop are i.i.d. RVs with PDF and CDF given in (15) and (16), respectively. The CDF $F_{\gamma_{e}}\left(\gamma \right)$ of the equivalent SNR $\gamma_{e}$ is given by

(25) $$\begin{array}{cc} F_{\gamma_{e}}\left(\gamma \right) & =\mathrm{Pr}\left[\gamma_{e}\leq \gamma \right]=1-\mathrm{Pr}\left[\gamma_{e}>\gamma \right]\\ & =1-\mathrm{Pr}\left[\min\left\{\gamma_{1},\ldots ,\gamma_{L}\right\}>\gamma \right]=1-\prod_{\ell =1}^{L}\mathrm{Pr}\left(\gamma_{\ell }>\gamma \right)\\ & =1-\prod_{\ell =1}^{L}\left[1-\mathrm{Pr}\left(\gamma_{\ell }\leq \gamma \right)\right]=1-\prod_{\ell =1}^{L}\left[1-F_{\gamma_{\ell }}\left(\gamma \right)\right]\\ & =1-{\left[\hat{\Gamma }\left(m,\frac{mN_{0}}{\Omega E_{b}P_{Rx,\ell +1}}\gamma \right)\right]}^{L}, \end{array}$$

where $\hat{\Gamma }\left(\cdot ,\cdot \right)$ is the normalized upper incomplete gamma function defined as

(26) $$\hat{\Gamma }\left(\tau ,y\right)=\frac{1}{\Gamma (\tau )}\int_{y}^{\infty }t^{\tau -1}\cdot \exp\left(-t\right)\;dt.$$

The PDF $f_{\gamma_{e}}\left(\gamma \right)$ of the equivalent SNR $\gamma_{e}$ follows immediately by differentiating (25) and is expressed as in (24). ☐

### 4.1. The End-to-End Average Capacity

The end-to-end average channel capacity $C_{avg}$ of the relay network can be obtained from

(27) $$C_{avg}=\int_{0}^{\infty }C\left(\gamma_{e}\right)\cdot f_{\gamma_{e}}\left(\gamma_{e}\right)\;d\gamma_{e},$$

where $C(\gamma_{e})$ is the instantaneous capacity in (23) and $f_{\gamma_{e}}\left(\cdot \right)$ is the PDF of the equivalent SNR $\gamma_{e}$ given in (24). The closed-form expression for the average capacity is given below.

**Proposition** **4.** *The end-to-end average capacity of the L-hop relay network under the balanced power allocation scheme can be expressed as follows:*

(28) Cavg=∑n=0∞∑q=1∞∑k=02q−1exp(−A)·Anln(2)n!·2·(−1)k2q−1·2q−1k·E2βnγe+2βnk.

*where the k-th moments $E\left[{\left(\frac{2\beta_{n}}{\gamma_{e}+2\beta_{n}}\right)}^{k}\right]$ of $\frac{2\beta_{n}}{\gamma_{e}+2\beta_{n}}$ can be expressed as follows*

(29) $$E\left[{\left(\frac{2\beta_{n}}{\gamma_{e}+2\beta_{n}}\right)}^{k}\right]=\sum_{\nu =0}^{\left(L-1)(m-1\right)}\frac{\omega_{\nu }\cdot \Gamma \left(m+v\right)}{\Gamma \left(m\right)\cdot L^{\frac{m+v-k-1}{2}}}\cdot {\left(\frac{2\beta_{n}mN_{0}}{\Omega E_{b}P_{Rx,\ell +1}}\right)}^{\frac{m+v+k-1}{2}}\cdot \exp\left(\frac{\beta_{n}mLN_{0}}{\Omega E_{b}P_{Rx,\ell +1}}\right),$$

*with $\omega_{0}=1$, $\omega_{1}=L-1$, $\omega_{\left(L-1)(m-1\right)}=\frac{1}{{\left[\left(m-1\right)!\right]}^{L-1}}$, and $\omega_{\nu }=\frac{1}{\nu }\sum_{\lambda =1}^{\Lambda }\frac{\lambda L-\nu }{\lambda !}\omega_{\nu -\lambda },\nu =2,3,\ldots ,\left(L-1\right)\left(m-1\right)-1$ are computed recursively with Λ=min{ν,m−1}.*

**Proof.**  See Appendix B.1. ☐

### 4.2. Statistics of the End-to-End Instantaneous Capacity

The expression of the end-to-end instantaneous capacity $C(\gamma_{e})$ of the multi-hop relay network is given in (23). To obtain the statistics of $C(\gamma_{e})$, we define an auxiliary variable $C_{n}^{*}$ called partial channel capacity as follows:

(30) $$\begin{array}{cc} C_{n}^{*}\left(\gamma_{e}\right) & =\frac{\alpha_{n}}{\ln(2)}\cdot \ln\left(1+\frac{\gamma_{e}}{\beta_{n}}\right) \end{array}$$

(31) $$\begin{array}{cc} C(\gamma_{e}) & =\sum_{n=0}^{\infty }C_{n}^{*}\left(\gamma_{e}\right)\approx \sum_{n=0}^{N}C_{n}^{*}\left(\gamma_{e}\right), \end{array}$$

where *N* is calculated from (13). With the assumptions in Section 2, it is straightforward to show that the auxiliary variables $C_{n}^{*}$ (n=0,1,…,N) are mutually independent.

Next, by applying the concept of transformation of random variables on (30) ([45], pp. 100–108), the PDF $f_{C_{n}^{*}}\left(c\right)$ of the variable $C_{n}^{*}$ (n=0,1,…,N) can be obtained from

(32) $$f_{C_{n}^{*}}\left(c\right)=f_{\gamma_{e}}\left(\gamma \right)\frac{d\gamma_{e}}{dC_{n}^{*}}{|}_{\gamma_{e}=C_{n}^{*-1}\left(c\right)}=\frac{\ln\left(2\right)\cdot \beta_{n}\cdot 2^{\frac{c}{\alpha_{n}}}}{\alpha_{n}}\cdot f_{\gamma_{e}}\left(\beta_{n}\cdot 2^{\frac{c}{\alpha_{n}}}-\beta_{n}\right),$$

where the function $f_{\gamma_{e}}\left(\cdot \right)$ is expressed in (24), $\alpha_{n}$ and $\beta_{n}$ are given in (8) and (10), respectively.

Finally, it is known that the PDF of the sum of multiple independent random variables, each of which has a PDF, is the convolution of their separate density functions ([45], pp. 182–186). Therefore, from (31), the PDF $f_{C}\left(c\right)$ of the end-to-end instantaneous channel capacity *C* is expressed as

(33) $$\begin{array}{cc} f_{C}\left(c\right) & =\left(f_{C_{0}^{*}}*\ldots *f_{C_{N}^{*}}\right)\left(c\right)\\ & =\underset{N-\mathrm{fold}}{\underset{︸}{\int ...\int }}f_{C_{N}^{*}}\left(c_{N}\right)\cdot f_{C_{N-1}^{*}}\left(c_{N-1}\right)\ldots f_{C_{1}^{*}}\left(c_{1}\right)\cdot f_{C_{0}^{*}}\left(c-c_{N}-c_{N-1}\ldots c_{1}\right)\;dc_{1}dc_{2}\ldots dc_{N}, \end{array}$$

where ∗ denotes the convolution operator, and the expression of the PDFs $f_{C_{n}^{*}}\left(c\right)$ (n=0,1,…,N) is given in (32) and (13). There exists no closed-form solution to the convolution in (33), but it can be numerically evaluated using mathematical softwares such as Mathematica and Matlab.

The variance of the channel capacity is a measurement of the spread of the instantaneous capacity around the average capacity. The variance of the end-to-end instantaneous capacity, denoted as $\sigma_{C}$, is defined as

(34) $$\sigma_{C}=\int_{0}^{\infty }{\left(c-C_{avg}\right)}^{2}\cdot f_{C}\left(c\right)\;dc,$$

where $C_{avg}$ is the end-to-end average capacity given in (A9), and $f_{C}\left(c\right)$ is the PDF of the end-to-end instantaneous capacity expressed in (33). Closed-form analytical expression for the variance of the channel capacity given in (34) is very difficult to obtain. Nevertheless, the result can be obtained numerically, as will be presented in Section 5.2.

## 5. Numerical Results

In this section, we will present and discuss the analytical results obtained in the previous sections. The validity of the theoretical results is confirmed with Monte Carlo simulations.

### 5.1. Results on Average BER

**Figure 5:**
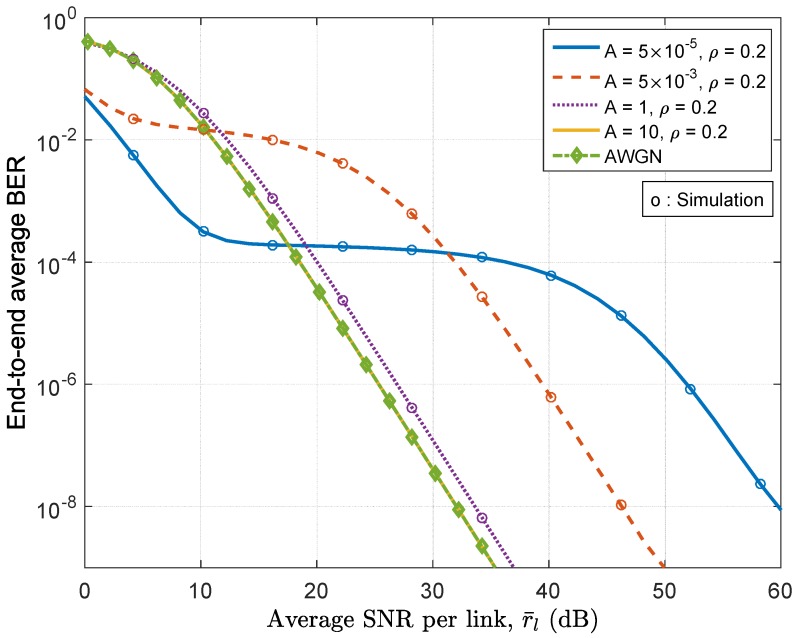
End-to-end average BER vs. average SNR per link over Nakagami-*m* (m=3) channels with various noise conditions.

In Figure 5, we present the end-to-end average BER for BPSK modulation signals of an 8-hop relay system against the average SNR of each hop over Nakagami-*m* fading channels with m=3 in the presence of impulsive noise with various levels of impulsiveness (i.e., from highly impulsive with $A=5\times 10^{-5}$, moderately impulsive with $A=5\times 10^{-3}$ to rarely impulsive with A=1,10, the ratio ρ is fixed as 0.2). The theoretical curves are obtained using the closed-form expressions given by (A5) and (18) and are found to agree well with the simulation results, thus validating our analysis. It can be seen that when the parameter *A* is equal to 10, the corresponding curve is extremely close to the result obtained under AWGN assumption, which is in accordance with our statement that when *A* is equal to or greater than 10, the MCA channel degenerates to an AWGN channel. It can also be observed from Figure 5 that the performance under impulsive noise is dramatically different from that under the AWGN. Under a highly impulsive noise condition, a three-region performance behavior is observed: the BER curve first decreases almost linearly with increasing SNR, and then remains almost stagnant for some SNR region until finally decreases again after increasing the SNR to a larger value. This phenomenon can be best explained by the envelop distribution analyses of the MCA interferences in [43]. It has been observed that the distributions of the noise envelop *z* are divided into three parts, the first corresponds to smaller values of *z*, in which the Gaussian noise component dominates; the second corresponds to larger values of *z*, wherein the impulsive noise component dominates; and the third corresponds to intermediate values of *z*, for which the CDF is virtually constant for these values. The portion of the distribution corresponding to these intermediate values of *z* is termed the “null region” [43]. Therefore, we can conclude that the first decrease in the BER plot is mainly affected by the Gaussian noise component with small envelopes; and the second decrease is mainly determined by the impulsive noise component while the stagnant flat-region in the BER plot is due to the existence of the “null region” in the envelope distribution of the MCA noise.

**Figure 6:**
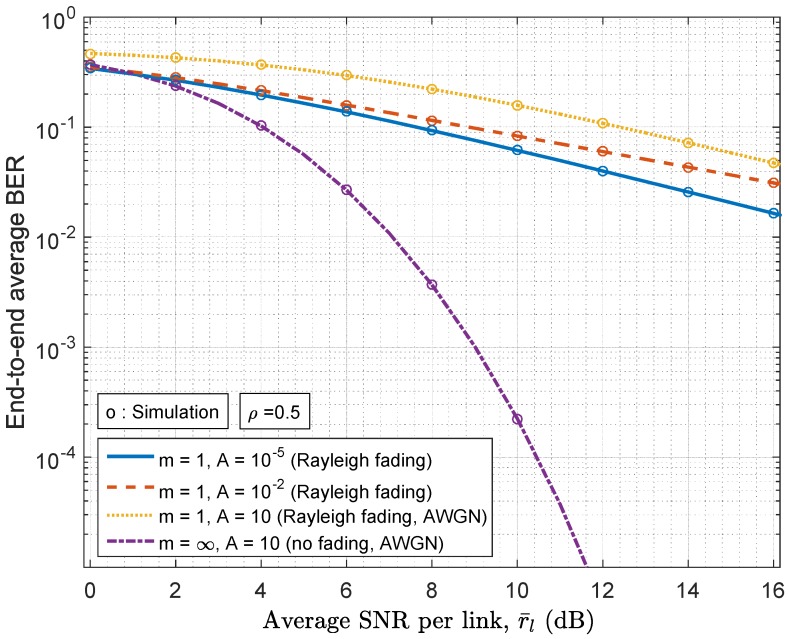
End-to-end average BER v.s. average SNR per channel in the scenarios of Rayleigh fading (m=1) and no fading (m=∞).

An advantage of our analysis lies in its flexibility for the performance evaluation under various channel conditions. For illustration purposes, Figure 6 shows the BER results of the Rayleigh fading channels in various noise conditions and the purely AWGN channel by setting the appropriate Nakagami-*m* and MCA parameters.

**Figure 7:**
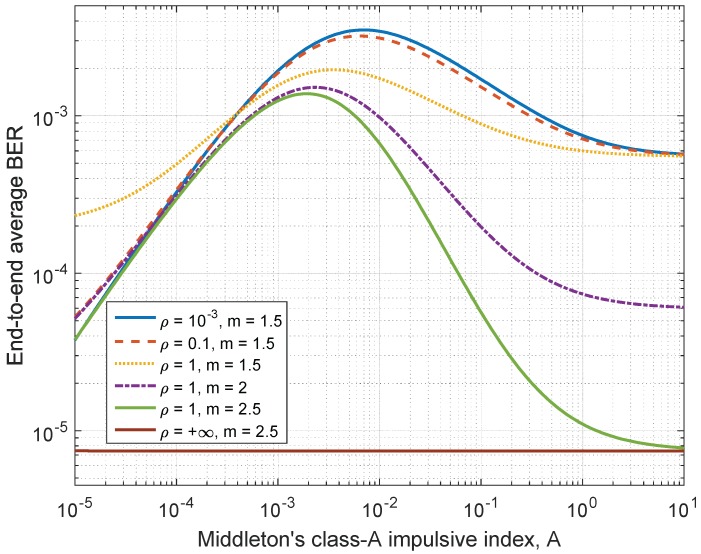
End-to-end average BER vs. MCA impulsive index *A* for various values of ρ and the Nakagami *m* parameter.

The effects of the impulsive index *A* and the ratio ρ on the end-to-end BER performance of the multi-hop relay network are investigated in Figure 7. It shows the average BER under various values of *A* and ρ for an 8-hop relay network with average SNR being 25 dB. It is observed that the BER is not a monotonic function of the impulsive index *A* with fixed values of ρ. When the channel noise changes from highly impulsive $\left(A=10^{-5}\right)$ to approximately Gaussian (A=10), the BER first increases for some range of *A* before reaching a peak point; then with further increase of *A* to around 10, the BER gradually decrease to the value under AWGN channel. The value of ρ=∞ corresponds to the case of purely AWGN channel, thus the BER value does not depend on *A* in this case and is always equal to the value of AWGN case.

**Figure 8:**
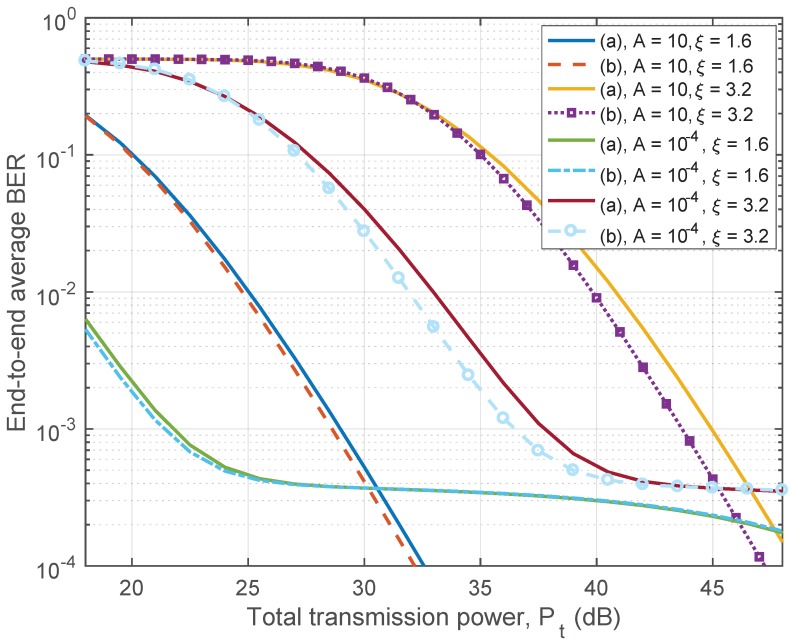
End-to-end average BER vs. total transmission power for different values of MCA impulsive index *A* and path-loss exponent ξ with (**a**) equal power allocation; (**b**) allocation scheme under (4).

In Figure 8, we present the end-to-end average BER performance of a relay network against total power consumption under two different power allocation schemes, i.e., the scheme of equal power allocation among all transmitting nodes, and the scheme of balanced power allocation taken into consideration the path loss of each hop. The following parameters are used for the simulation: L=8, m=3, ρ=0.2, $\left(\Omega \cdot E_{b}\right)/N_{0}=10$; and the distances of each hop is set as $d_{\left\{1,\ldots ,8\right\}}=\left\{8.2,6.1,11.3,7.2,8.5,6.8,6.9,9.8\right\}$ meters. The BER results under the equal power allocation scheme are calculated recursively using (A4). It can be seen that the performance under balanced power allocation scheme is generally better than that under the equal power allocation scheme. Also, the performance difference between the two schemes becomes larger when the path loss exponent is greater. This is because under the balanced power allocation scheme, the performances of all hops are always identical; while for the equal power allocation scheme, when the path loss exponent is larger, the variance of the performances of each hop also becomes larger. This eventually leads to poorer performance of the relaying system since the end-to-end performance of the DF relaying network is dominated by the performance of the worst hop.

### 5.2. Results on Channel Capacity

**Figure 9:**
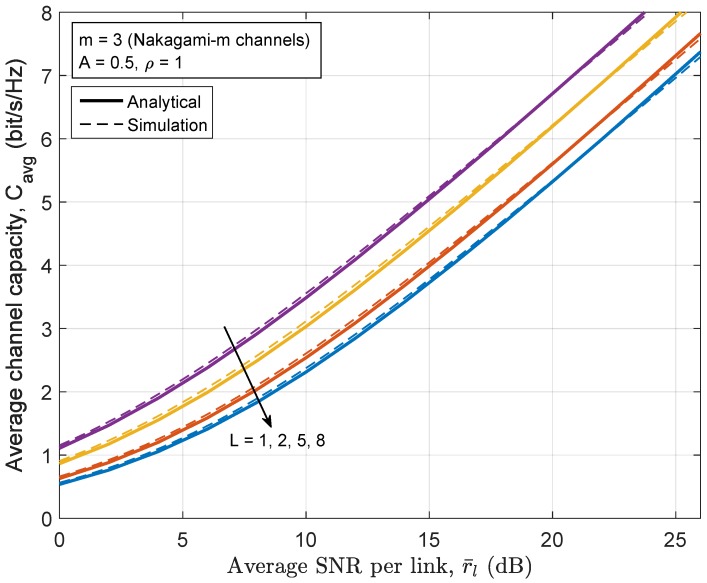
The channel capacity of Nakagami-*m* channels (m=3) with L=1,2,5,8 relay hops.

**Figure 10:**
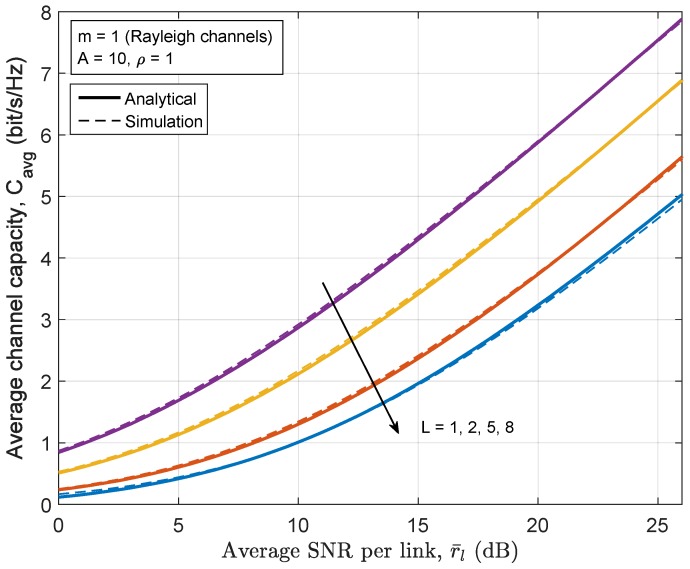
The channel capacity of Rayleigh channels (m=1) and nearly AWGN (A=10) with L=1,2,5,8 relay hops.

Figure 9 and Figure 10 sketch the end-to-end average capacity of the relay network employing the full-duplex communication mode and balanced power allocation scheme as a function of the average SNR for varying numbers of hops *L*. It can be seen that excellent fit is found between the analytical results from the proposed closed-form expression and the simulation results. Figure 9 shows the channel capacity of relay network under the Nakagami-*m* fading channels (m=3) in the presence of mediumly impulsive noise with A=0.5 and ρ=1. Figure 10 shows the channel capacity of the relay network under Rayleigh fading channels (m=1) in the presence of nearly Gaussian noise with A=10. As expected, the overall channel capacity degrades as *L* increases in exchange for broader transmission coverage. A less impulsive channel (i.e., when *A* or ρ are large enough such that the MCA channel degenerates to the AWGN channel) gives a capacity approaching that of the AWGN channel. Since AWGN is known to be the worst additive interference in terms of channel capacity for both point-to-point channels and relay channels, all values of the different MCA model parameter sets provide higher capacity than the corresponding AWGN channel [48]. However, it should be noted that the AWGN channel does not necessarily underperform the corresponding MCA channel in terms of BER, as can be seen from Figure 7.

**Figure 11:**
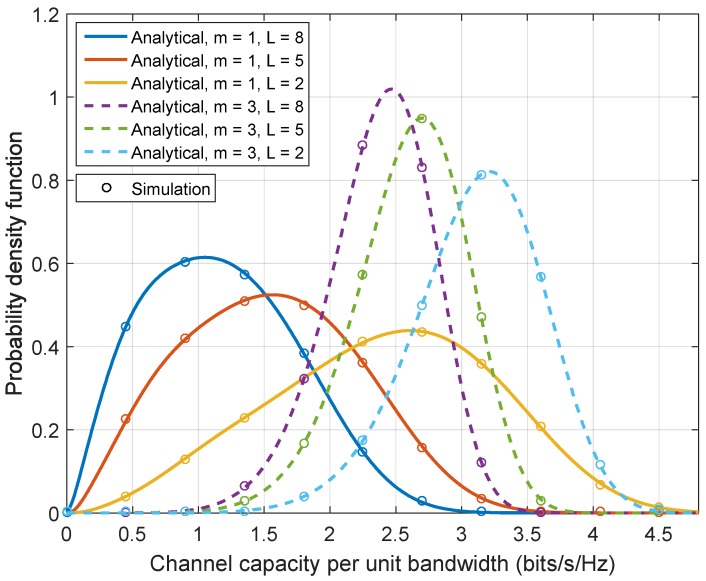
The PDF of channel capacity of Rayleigh channels (m=1) and Nakagami-*m* channels (m=3) with L=8,5,2 relay hops.

**Figure 12:**
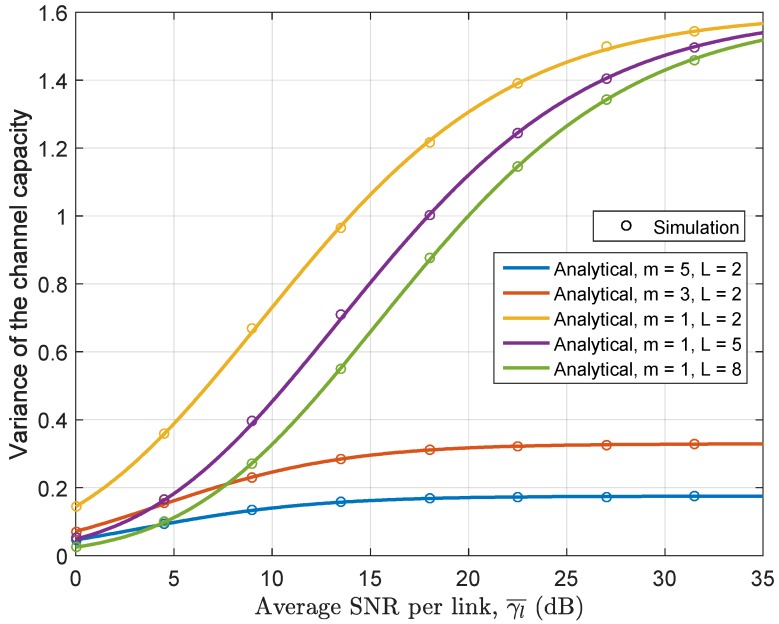
The variance of channel capacity for the relay fading channels with different values of *m* and *L*, A=0.5,ρ=1.

The PDFs of the end-to-end instantaneous capacity of the multi-hop relay system under Rayleigh (m=1) and Nakagami-*m* (m=3) fading channels are presented in Figure 11. The following parameters are considered: the impulsive index A=0.5, the ratio ρ=1, and the average SNR being 10 dB. It can be found from Figure 11 that an increase of the severity of large-scale fading (i.e., decreasing the value of the Nakagami-*m* parameter *m*) decreases the mean value of the channel capacity. Similarly, an increase of the number of hops *L* also degrades the mean channel capacity. These can also be evidenced by comparing Figure 9 and Figure 10. It can also be observed that a decrease in the parameter *m* or the number of hops *L* results in an increase of the variance of the channel capacity, which will be reconfirmed in Figure 12.

Figure 12 illustrates the behavior of the variance of the channel capacity with varying average SNR per link. We calculate the variance for different number of hops and varying values of the Nakagami *m* parameter. It is shown that the variance increases as the average SNR increases. More specifically, it is found that the variance increase monotonically from low SNR to high SNR region and afterward it continues to maintain approximately the same level in the high SNR region. The variation of the channel capacity is also found to increase with the reduction of the Nakagami *m* parameter or increase of the number of relay hops.

## 6. Conclusions

In this paper, we investigated the end-to-end average BER performance and the channel capacity of a multi-hop DF relay system over Nakagami-*m* fading in the presence of impulsive noise. The analysis were validated by showing the excellent agreement between the results obtained through the closed-form expressions and the Monte Carlo simulation results. The analysis is very general and can be easily extended to other channel conditions (e.g., the conventional Rayleigh fading over AWGN, etc.) by changing the parameters of the fading and the noise models. The impacts of the path loss exponent and the fading severity on the system performance are also investigated. It is shown that the increase of the number of hops will degrade both the BER and channel capacity but also decrease the variance of the channel capacity. In channel conditions with highly “impulsive” noise effects, the BER curve will experience a stagnant stage against the increase of the SNR due to the characteristics of the noise. As the path loss exponent of the propagation environment increases, the advantage of the balanced power allocation scheme becomes more obvious compared to the equal power allocation scheme with respect to the end-to-end BER.

Future work includes improvement of system performance under impulsive noise conditions by noticing that the existence of impulsive noise significantly degrades the performance, especially in the “null region”. This can be potentially achieved by predicting the occurrence of impulsive noise if possible and adjust the power or coding rate accordingly.

## Appendix A. Bit Error Rate Performance Analysis

### Appendix A.1. Derivation for the Average BER P_ℓ_ in Proposition 1

Let $P_{\ell }\left(e|\gamma \right)$ be the conditional BER of the *ℓ*-th hop conditioned on SNR γ. By interpreting the MCA channel as a Gaussian-mixture model, the BER for M-ary PSK signals as a function of the SNR can be expressed as [49]

(A1) $$P_{\ell }\left(e|\gamma \right)=\sum_{n=0}^{\infty }\alpha_{n}\cdot \frac{2}{\zeta_{M}}\sum_{\iota =1}^{\max(\frac{M}{4},1)}Q\left(\sqrt{\frac{A\gamma (1+\rho )}{\left(n+A\rho \right)}}\cdot \eta_{\iota }\right),$$

where $\zeta_{M}=\max\left(\log_{2}M,2\right)$, $\eta_{\iota }=\sin\left(\frac{(2\iota -1)\cdot \pi }{M}\right)$, $\alpha_{n}$ is given in (8) and Q(·) is the Gaussian Q-function.

The average BER $P_{\ell }$ of the *ℓ*-th hop over Nakagami-*m* fading in the presence of impulsive noise is given by

(A2) $$\begin{array}{cc} P_{\ell } & =\int_{0}^{\infty }P_{\ell }\left(e|\gamma \right)\cdot f_{\gamma_{l}}\left(\gamma \right)\;d\gamma =\sum_{n=0}^{\infty }\alpha_{n}\cdot \frac{{\left(mN_{0}\right)}^{m}}{{\left(\Omega E_{b}P_{Rx,\ell +1}\right)}^{m}\Gamma \left(m\right)}\cdot \frac{2}{\zeta_{M}}\sum_{\iota =1}^{max(\frac{M}{4},1)}\cdot \int_{0}^{\infty }\gamma^{m-1}\\ & \cdot Q\left(\sqrt{\frac{A\gamma (1+\rho )}{\left(n+A\rho \right)}}\cdot \eta_{\iota }\right)\cdot \exp\left(-\frac{m\gamma N_{0}}{\Omega E_{b}P_{Rx,\ell +1}}\right)\;d\gamma . \end{array}$$

The indefinite integral in above expression is in the form of $I\left(m,\mu_{1},\mu_{2}\right)=\int_{0}^{\infty }\gamma^{m-1}\cdot Q\left(\mu_{1}\sqrt{\gamma }\right)\cdot \exp\left(-\mu_{2}\gamma \right)\;d\gamma$, which has the following solution [50]:

(A3) I(m,μ1,μ2)=u2m·Γ(m)2−u122π·Γ(m+12)u2+u122m+12·F121,m+12;32;u12u12+2u2,

where _2_$F_{1}\left(a,b;c;x\right)$ is the Gauss hypergeometric function.

Utilizing (A3) in (A2), we obtain the closed-form expression of the average BER $P_{\ell }$ of the *ℓ*-th hop as shown in (18) and (19).

### Appendix A.2. Derivation for the BER Relationship in Proposition 2

In the investigated DF relay network, the transmitted information-bearing signal can be correctly decoded at the destination node if the relay performs correct detection, or if a relay makes an erroneous detection, which is rectified by further erroneous detection at subsequent relays [15]. Let $P_{\ell }^{E}$ denotes the BER after the *ℓ*-th hop transmission starting from the source node. Then, the end-to-end average BER $P_{L}^{E}$ for the wireless relay system can be written as

(A4) $$\begin{array}{cc} P_{L}^{E} & =P_{L}\left(1-P_{L-1}^{E}\right)+P_{L-1}^{E}\left(1-P_{L}\right)\\ & =P_{L}-P_{L-1}^{E}\left(2P_{L}-1\right), \end{array}$$

where $P_{L}$ is the average BER of the *L*-th hop transmission and can be calculated from (18).

If the channel conditions are identical for all hops and transmission power of each hop fulfils the constraint in (4), the average BER of all individual hops are equal (denoted as $P_{0}$). Then, we can extend the expression (A4) recursively and obtain the following relationship between the end-to-end average BER and the average BER of individual hops:

(A5) $$\begin{array}{cc} P_{L}^{E} & =P_{0}-P_{L-1}^{E}\left(2P_{0}-1\right)\\ & =P_{0}-\left[P_{0}-P_{L-2}^{E}\left(2P_{0}-1\right)\right]\left(2P_{0}-1\right)\\ & =P_{0}-P_{0}\left(2P_{0}-1\right)+P_{L-2}^{E}{\left(2P_{0}-1\right)}^{2}\\ & =\ldots \ldots \\ & =P_{0}+P_{0}\left(1-2P_{0}\right)+\ldots +P_{1}^{E}{\left(1-2P_{0}\right)}^{L-1}\\ & =\sum_{\ell =0}^{L-1}P_{0}{\left(1-2P_{0}\right)}^{\ell }=\frac{1}{2}\left[1-{\left(1-2P_{0}\right)}^{L}\right], \end{array}$$

where the last equality is obtained based on the properties of geometric progression ([46], p. 1).

## Appendix B. Channel Capacity Performance Analysis

### Appendix B.1. Derivation of the Average Capacity in Proposition 4

To derive the closed-form expression for average capacity, we first obtain the following alternative expression of the logarithm term in (23) by expanding it to Taylor series ([51], p. 68):

(A6) $$\begin{array}{cc} \ln\left(1+\frac{\gamma_{e}}{\beta_{n}}\right) & =\sum_{q=1}^{\infty }\frac{2}{2q-1}{\left(1-\frac{2\beta_{n}}{\gamma_{e}+2\beta_{n}}\right)}^{2q-1} \end{array}$$

(A7) =∑q=1∞∑k=02q−122q−12q−1k·−2βnγe+2βnk.

Substituting (A7) and (23) into (27), we can obtain the following expression for the end-to-end average capacity $C_{avg}$ of the multi-hop network:

(A8) Cavg=∑n=0∞αnln(2)·∫0∞∑q=1∞∑k=02q−122q−1·2q−1k·−2βnγe+2βnk·fγe(γe)dγe

(A9) =∑n=0∞∑q=1∞∑k=02q−1exp(−A)·Anln(2)n!·2·(−1)k2q−1·2q−1k·E2βnγe+2βnk.

It is obvious from (A9) that to calculate the average capacity, we need to derive the moments of the ratio $\frac{2\beta_{n}}{\gamma_{e}+2\beta_{n}}$. By definition, the *k*-th moment $E[{\left(\frac{2\beta_{n}}{\gamma_{e}+2\beta_{n}}\right)}^{k}]$ is formulated as

(A10) $$E\left[{\left(\frac{2\beta_{n}}{\gamma_{e}+2\beta_{n}}\right)}^{k}\right]=\int_{0}^{\infty }{\left(\frac{2\beta_{n}}{\gamma +2\beta_{n}}\right)}^{k}\cdot f_{\gamma_{e}}\left(\gamma \right)\;d\gamma =\frac{L}{\Gamma (m)}\cdot {\left(\frac{mN_{0}}{\Omega E_{b}P_{Rx,\ell +1}}\right)}^{m}\cdot I_{a},$$

where the integral $I_{a}$ is given as

(A11) $$I_{a}=\int_{0}^{\infty }\gamma^{m-1}\cdot {\left(\frac{2\beta_{n}}{\gamma +2\beta_{n}}\right)}^{k}\cdot \exp\left(-\frac{mN_{0}\gamma }{\Omega E_{b}P_{Rx,\ell +1}}\right)\cdot {\left[\hat{\Gamma }\left(m,\frac{mN_{0}\gamma }{\Omega E_{b}P_{Rx,\ell +1}}\right)\right]}^{L-1}d\gamma .$$

To obtain the closed-form expression for integral $I_{a}$ in (A10), we follow the similar rationale in [52], namely, representing the Gamma function as a sum of terms. The normalized upper incomplete Gamma function can be rewritten as an finite sum of terms ([46], p. 899), i.e.,

(A12) $$\hat{\Gamma }\left(m,\frac{mN_{0}\gamma }{\Omega E_{b}P_{Rx,\ell +1}}\right)=\exp\left(\frac{-mN_{0}\gamma }{\Omega E_{b}P_{Rx,\ell +1}}\right)\cdot \left[\sum_{\nu =0}^{m-1}\frac{1}{\nu !}{\left(\frac{mN_{0}}{\Omega E_{b}P_{Rx,\ell +1}}\gamma \right)}^{\nu }\right].$$

Next, we define $\varphi =\left(mN_{0}\gamma \right)/\left(\Omega E_{b}P_{Rx,\ell +1}\right)$ and $\psi_{\nu }=1/\left(\nu !\right)$. According to the polynomial theory, the finite series in (A12) corresponds to a polynomial $Q_{1}$ in φ with degree (m−1) and coefficient $\psi_{\nu }$ ([46], p. 17). Then, the (L−1)-th power of $Q_{1}$ is equivalent to another polynomial $Q_{2}$ with degree (L−1)(m−1) as follows

(A13) $${\left[\sum_{\nu =0}^{m-1}\psi_{\nu }\cdot \varphi^{\nu }\right]}^{L-1}=\sum_{\nu =0}^{\left(L-1)(m-1\right)}\omega_{\nu }\cdot \varphi^{\nu },$$

where the coefficients $\omega_{\nu }$ are expressed as

(A14) $$\omega_{0}=1,\;\omega_{1}=L-1,\;\omega_{\left(L-1)(m-1\right)}=\frac{1}{{\left[\left(m-1\right)!\right]}^{L-1}},$$

and

(A15) $$\omega_{\nu }=\frac{1}{\nu }\sum_{\lambda =1}^{\Lambda }\frac{\lambda L-\nu }{\lambda !}\omega_{\nu -\lambda },\;\nu =2,3,\ldots ,\left(L-1\right)\left(m-1\right)-1$$

are computed recursively with Λ=min{ν,m−1}.

Using (A12) and (A13) for the integral $I_{a}$ in (A10) and applying the equality [46, Eq. 3.384.3], we can obtain the exact closed-form solution for the integral $I_{a}$ as

(A16) $$\begin{array}{cc} I_{a}= & \sum_{\nu =0}^{\left(L-1)(m-1\right)}\;\;\;\;\;\omega_{\nu }\cdot {\left(\frac{mN_{0}}{\Omega E_{b}P_{Rx,\ell +1}}\right)}^{\nu }\cdot \int_{0}^{\infty }\gamma^{m+v-1}{\left(\frac{2\beta_{n}}{\gamma +2\beta_{n}}\right)}^{k}\cdot \exp\left(-\frac{mLN_{0}}{\Omega E_{b}P_{Rx,\ell +1}}\gamma \right)\;d\gamma \end{array}$$

(A17) $$\begin{array}{cc} = & \sum_{\nu =0}^{\left(L-1)(m-1\right)}\omega_{\nu }\cdot \frac{\Gamma (m+v)}{L^{\frac{m+v-k+1}{2}}}\cdot {\left(\frac{\Omega E_{b}P_{Rx,l+1}}{mN_{0}}\right)}^{\frac{m-v-k+1}{2}}\cdot {\left(2\beta_{n}\right)}^{\frac{m+v+k-1}{2}}\cdot \exp\left(\frac{\beta_{n}mLN_{0}}{\Omega E_{b}P_{Rx,\ell +1}}\right). \end{array}$$

Finally, the *k*-th moments $E\left[{\left(\frac{2\beta_{n}}{\gamma_{e}+2\beta_{n}}\right)}^{k}\right]$ of $\frac{2\beta_{n}}{\gamma_{e}+2\beta_{n}}$ can be expressed as follows

(A18) $$E\left[{\left(\frac{2\beta_{n}}{\gamma_{e}+2\beta_{n}}\right)}^{k}\right]=\sum_{\nu =0}^{\left(L-1)(m-1\right)}\frac{\omega_{\nu }\cdot \Gamma \left(m+v\right)}{\Gamma \left(m\right)\cdot L^{\frac{m+v-k-1}{2}}}\cdot {\left(\frac{2\beta_{n}mN_{0}}{\Omega E_{b}P_{Rx,\ell +1}}\right)}^{\frac{m+v+k-1}{2}}\cdot \exp\left(\frac{\beta_{n}mLN_{0}}{\Omega E_{b}P_{Rx,\ell +1}}\right),$$

where the parameters $\omega_{\nu }$ are given in (A14) and (A15).

Finally, the end-to-end average capacity of the *L*-hop relay network can be obtained by substituting the expressions of the term $E\left[{\left(\frac{2\beta_{n}}{\gamma_{e}+2\beta_{n}}\right)}^{k}\right]$ given in (A18) into (A9).
